# Associations of Food Neophobia and Food Choice Motives with Organic Food Consumption Orientation Among Adults in Türkiye: A Cross-Sectional Study

**DOI:** 10.3390/foods15203637

**Published:** 2026-10-13

**Authors:** Muhammet Ali Çakır, Merve Pehlivan, Neslişah Denkçi, Perim Fatma Türker

**Affiliations:** 1 Department of Nutrition and Dietetics, Faculty of Health Sciences, Kırklareli University, 39000 Kırklareli, Türkiye; cakir34@gmail.com; 2 Department of Nutrition and Dietetics, Faculty of Health Sciences, Yuksek Ihtisas University, 06520 Ankara, Türkiye; merve.pehlivan@yuksekihtisas.edu.tr; 3 Department of Nutrition and Dietetics, Institute of Health Sciences, Trakya University, 22030 Edirne, Türkiye; denkcineslisah@gmail.com; 4 Department of Nutrition and Dietetics, Graduate School of Health Sciences, Acibadem Mehmet Ali Aydinlar University, 34752 Istanbul, Türkiye; 5 Department of Nutrition and Dietetics, Faculty of Health Sciences, Acibadem Mehmet Ali Aydinlar University, 34752 Istanbul, Türkiye

**Keywords:** organic food consumption orientation, food neophobia, food choice motives, psychosocial determinants, dietary behaviour, sustainable diets, adults, public health

## Abstract

Organic food consumption has gained increasing attention in the context of healthy and sustainable dietary behaviours. However, the roles of food neophobia and food choice motives remain insufficiently understood. Whether food neophobia and food choice motives were related to organic food consumption orientation, defined by the Organic Food Consumption Scale (OFCS), was evaluated in 506 adults aged 18–64 years from Kırklareli, Türkiye. Participants completed the OFCS, the Food Neophobia Scale and the Food Choice Questionnaire in a single session using a paper-based self-administered questionnaire. Associations were estimated with Spearman coefficients and multivariable linear models adjusted for age and gender. Overall, 73.5% reported consuming foods they considered organic. The total food choice motive score was positively associated with organic food consumption orientation, whereas the total food neophobia score was not. At the subdimension level, health, natural content (the strongest association) and weight control motives were positively associated with orientation. Within food neophobia, greater willingness to try new foods and lower trust in new foods were associated with a stronger orientation. Orientation was thus associated not with overall food neophobia but with its specific dimensions and with food choice motives. These findings may inform research on consumer orientations towards organic food.

## 1. Introduction

Interest in organic food consumption has increased considerably, alongside growing health and environmental awareness [1]. Organic foods are generally defined as foods produced through systems based, as much as possible, on natural inputs, physical, mechanical, or biological farming methods, and living conditions that allow animals to express their natural behaviours [2]. As a result, organically produced foods are often perceived by consumers as safer and of higher quality than conventionally produced alternatives [1]. This growing global interest has also increased scientific attention towards the factors that shape environmentally friendly and health-related food consumption behaviours [3]. Although research on organic food consumption continues to expand, the psychological and behavioural mechanisms underlying organic food purchasing intentions and their translation into actual behaviour have not yet been fully clarified [1,4].

One factor that may help explain this intention–behaviour gap is food neophobia. Food neophobia describes a tendency to avoid foods perceived as novel or unfamiliar, and it has been proposed as a factor that may shape the uptake of organic foods [4]. However, findings on the role of food neophobia in organic food consumption remain inconsistent. Some studies have reported that food neophobia does not affect willingness to try organic foods or preference for them, and that it does not play a moderating role in the attitude–intention relationship [5,6,7]. In contrast, other studies have shown that food neophobia may negatively affect organic food purchase intention and may moderate the intention–behaviour relationship [1,4,8]. Beyond the specific context of organic foods, food neophobia has also been associated with broader differences in dietary patterns, food choice motives, and food-related behaviours. This association suggests that food neophobia may shape how individuals evaluate and select foods in everyday life [9,10,11].

At the same time, increasing consumer interest in organic foods and the growing market potential have led researchers to examine the motivations underlying organic food consumption in greater detail. Organic foods are often differentiated from conventional foods by their abstract attributes, and this has been identified as one of the main reasons for increasing consumer demand [12]. Reported reasons for choosing organic foods span health, food safety and product quality as well as environmental and ethical considerations and support for local producers [13]. However, consumer preferences for organic foods appear to be highly heterogeneous. The literature generally distinguishes between two broad consumption orientations. The first is an individual or egoistic orientation related to personal benefits such as health, safety, quality, taste, freshness, naturalness, and nutritional value. The second is an ethical or altruistic orientation related to environmental protection, animal welfare, sustainable agriculture, and local development [14]. Nevertheless, there is still no clear consensus on whether altruistic or individual factors play a more dominant role in organic food consumption [15]. In addition, the relative importance of these motivations appears to differ between developed and developing countries [13].

Despite growing research on organic food consumption, three aspects remain insufficiently examined. First, previous studies in the organic food context have generally treated food neophobia as a single overall construct [4,5,6,7,8,10,11]. In several of these studies, food neophobia was examined as a direct predictor or moderator within intention- or behaviour-oriented models [4,7,8,10,11]. No study identified in the reviewed literature has examined whether the subdimensions of food neophobia are differentially associated with organic food consumption orientation. Second, limited research has examined the nine food choice motives of the Food Choice Questionnaire together with a multidimensional measure of organic food consumption orientation in the same adult sample. Third, existing evidence suggests that these associations may vary across sociocultural contexts, and further research is needed to determine whether such relationships are consistent across different populations [9,13]. Evidence from Türkiye, in particular, remains limited. Accordingly, this study investigated whether food neophobia and food choice motives were associated with organic food consumption orientation among adults recruited in Kırklareli, Türkiye, and addressed three research questions. RQ1: Are the total food neophobia score and its two subdimensions (willingness to try new foods and trust in new foods) associated with organic food consumption orientation? RQ2: Which food choice motives are associated with organic food consumption orientation, and do these associations persist after adjustment for age and gender? RQ3 (exploratory): Are the associations observed for the total Organic Food Consumption Scale (OFCS) score also observed for its subdimensions? No directional hypotheses were prespecified, because previous findings, particularly on food neophobia, have been inconsistent.

## 2. Materials and Methods

### 2.1. Study Design

A cross-sectional survey was conducted in the city centre of Kırklareli, Türkiye, between February and April 2024 and is reported in accordance with the STROBE recommendations for cross-sectional studies [16]. Data were collected using paper-based self-administered questionnaires. The researchers approached adults living in the city centre in person, informed them about the purpose of the study and invited them to participate. Those who agreed completed the questionnaire themselves in a single session lasting approximately 20–30 min, in the presence of a researcher who was available to provide clarification if requested. No payment or other incentive was offered for participation.

### 2.2. Population, Sample and Sampling Procedure

According to 2023 data of the Turkish Statistical Institute, the source population comprised 246,256 residents of Kırklareli aged 18–64 years [17]. The minimum sample size was calculated using G*Power 3.1.9.4 (Heinrich Heine University Düsseldorf, Düsseldorf, Germany) based on a two-tailed point-biserial correlation model, assuming 90% power, a 5% significance level, and a small effect size (|ρ| = 0.15), yielding a minimum sample of 459 participants [18,19]. To account for potential data loss, the recruitment target was increased by approximately 20% to 550 participants. The original sample-size calculation was based on the planned analysis of self-reported organic food consumption status; analyses using the continuous OFCS score were subsequently conducted to examine organic food consumption orientation.

A non-probability convenience sampling method was used [20]. The demographic composition of the sample is compared descriptively with the 2023 provincial population in Section Limitations and Future Directions. Adults aged 18–64 years who were able to complete the questionnaire and agreed to participate were eligible. Individuals who could not communicate adequately or who were following a special diet were not enrolled. Records were excluded from the analysis if the questionnaire was incomplete, if the reported age was outside the range of 18–64 years, or if self-reported weight or height values were judged implausible on inspection. Questionnaires were obtained from 550 individuals. Following data screening, 44 records were excluded because of incomplete responses that prevented calculation of the study scale scores (*n* = 33), a reported age outside the eligibility range of 18–64 years (*n* = 5), or self-reported weight or height values that were judged implausible on inspection (*n* = 6). Consequently, 506 participants were included in the final analysis.

### 2.3. Ethical Considerations

The study protocol was approved by the Scientific Research and Publication Ethics Committee of Kırklareli University Rectorate (23 January 2023; No. 75503). Participants were informed about the purpose of the study and gave written informed consent before taking part; participation was voluntary and responses were handled confidentially. The study followed the principles of the 1964 Declaration of Helsinki and its later amendments.

### 2.4. Data Collection Instruments

The questionnaire combined sociodemographic and lifestyle questions with three validated Turkish instruments: the OFCS, the T-FNS and the FCQ. The first part covered age, gender, marital status, educational level, occupation, income status, chronic disease, smoking and alcohol use, exercise habits, self-reported body weight and height, previous nutrition education and organic food consumption habits. Gender was self-reported using a single item with two response options (woman/man); biological sex was not assessed, and other gender identities could not be recorded with this item [21]. The OFCS assessed organic food consumption orientation, the T-FNS the tendency to avoid new or unfamiliar foods, and the FCQ the motives underlying food choice.

#### 2.4.1. Organic Food Consumption Scale (OFCS)

Organic food consumption orientation was measured with the OFCS, developed and validated in Türkiye by Eti İçli et al. [22]. The 18-item scale comprises six subdimensions: Norms (4 items), Health Consciousness (3), Self-Identity (3), Benefits of Organic Food Consumption (4), Positive Moral Attitudes (2) and Information Seeking (2). Items are rated from 1 (“strongly disagree”) to 5 (“strongly agree”), and higher scores reflect a stronger orientation towards organic food consumption. The scale does not measure the frequency of consumption. Throughout this article, organic food consumption orientation therefore refers to the OFCS score, whereas reported organic food consumption refers to participants’ answers to the direct questions on whether and how often they consume organic food. In the original validation study, subdimension Cronbach’s alpha values ranged from 0.70 to 0.89, composite reliabilities from 0.79 to 0.94, and confirmatory factor analysis yielded acceptable fit (χ^2^/df = 1.94; RMSEA = 0.039; CFI = 0.98) [22]. Factor analysis was not repeated in the present study.

#### 2.4.2. Food Neophobia Scale (T-FNS)

Food neophobia was assessed with the 10-item Food Neophobia Scale of Pliner and Hobden [23] in its Turkish adaptation [24]. Items are scored from 1 (“strongly disagree”) to 7 (“strongly agree”); items 1, 4, 6, 9 and 10 are reverse scored, and total scores range from 10 to 70, with higher scores indicating stronger neophobic tendencies. The Turkish version contains two subdimensions, Willingness to Try New Foods and Trust in New Foods, both scored in the direction of neophobia. A higher Willingness to Try New Foods score therefore indicates lower willingness to try new foods, and a higher Trust in New Foods score indicates lower trust in new foods. In the adaptation study, Cronbach’s alpha was 0.805 for the total scale and 0.769 and 0.733 for the two subdimensions, and confirmatory factor analysis indicated acceptable fit (CFI = 0.942; RMSEA = 0.075) [24].

#### 2.4.3. Food Choice Questionnaire (FCQ)

Food choice motives were measured with the 36-item Food Choice Questionnaire of Steptoe et al. [25] in its Turkish version [26], which covers nine subdimensions: health, mood, convenience, sensory appeal, natural content, price, weight control, familiarity and ethical concern. As in the original instrument, items are rated from 1 (“not at all important”) to 4 (“very important”). The Turkish adaptation followed translation and back-translation procedures and reported a Cronbach’s alpha of 0.90 for the total scale, subdimension values between 0.60 and 0.84, and test–retest intraclass correlation coefficients between 0.89 and 0.95 [26].

### 2.5. Anthropometric Measurements

Participants reported their own body weight and height following instructions given in the questionnaire, and body mass index (BMI) was derived as weight in kilograms divided by the square of height in metres. BMI was categorised according to the World Health Organization classification: <18.5 kg/m^2^ underweight, 18.5–24.9 kg/m^2^ normal weight, 25.0–29.9 kg/m^2^ overweight and ≥30.0 kg/m^2^ obese [27].

### 2.6. Statistical Analysis

Categorical variables were summarised as number and percentage, and continuous variables as mean ± standard deviation (SD) with minimum and maximum values. Because the scale scores departed from a normal distribution, medians with the first and third quartiles (Q1–Q3) were reported alongside means and SDs. Internal consistency of the scales was examined using Cronbach’s alpha, with 0.70 used as a conventional benchmark for acceptable internal consistency [28]. Distributional assumptions were checked with the Kolmogorov–Smirnov test. Because the scale scores were not normally distributed, bivariate associations were estimated with Spearman’s rank correlation, and coefficients were interpreted according to their absolute value as very weak (|r_s_| ≤ 0.25), weak (0.26–0.49), moderate (0.50–0.69), strong (0.70–0.89) or very strong (≥0.90) [29]. Multivariable linear regression with the enter method was then used to relate the total and subdimension scores of the T-FNS and FCQ to the OFCS total and subdimension scores. Models were fitted without adjustment and with age and gender as covariates, and explanatory power was summarised as adjusted R^2^ (Adj. R^2^). Analyses of the OFCS subdimension scores were considered exploratory. Multicollinearity was evaluated with tolerance and variance inflation factor (VIF) values, with tolerance below 0.20 or VIF above 5 taken to indicate a problem. Regression assumptions for the two primary age- and gender-adjusted models of the total OFCS score were evaluated using residual-versus-fitted and Q–Q plots, the Shapiro–Wilk test for residual normality [30], the Koenker studentised modification of the Breusch–Pagan test for heteroscedasticity [31,32], Cook’s distance for influential observations, and standardised residuals for outlying observations [33]. Where heteroscedasticity was detected, heteroscedasticity-consistent HC3 standard errors were computed as a sensitivity analysis [34]. Residual-versus-fitted and normal Q–Q plots for these adjusted models are provided in Figure S1. All analyses were performed using SPSS version 26.0 (IBM Corp., Armonk, NY, USA). Two-sided *p* values < 0.05 were considered statistically significant.

## 3. Results

Participants had a mean age of 36.47 ± 12.05 years (median 35, Q1–Q3 26–45); 61.3% were women, 96.4% lived in urban areas, 57.1% were married, and 54.3% had completed higher education. Most participants (66.6%) were employed, and 74.1% described their income as sufficient. Organic food consumption was reported by 73.5%, with 37.2% consuming organic food more than once a week. An organic label or logo was the most commonly reported criterion for identifying organic food (54.2%), whereas 26.9% reported no specific criterion; this proportion was 15.1% among those who reported consuming organic food and 59.7% among those who did not (Table 1).

Mean total scores were 40.78 ± 8.81 for the T-FNS, 97.07 ± 17.33 for the FCQ and 61.62 ± 12.20 for the OFCS; medians, subdimension scores and internal consistency estimates are presented in Table 2. When expressed as mean item scores to account for differences in the number of items across subdimensions, Sensory Appeal had the highest FCQ score (3.23), whereas Ethical Concern (2.57) and Weight Control (2.69) had the lowest scores. Among the OFCS subdimensions, Benefits of Organic Food Consumption had the highest mean item score (3.84) and Norms the lowest (2.81). Cronbach’s alpha was 0.925 for the OFCS total score, ranged from 0.828 to 0.924 across its subdimensions, and was 0.939 for the FCQ total score. The T-FNS total score (α = 0.643), Trust in New Foods (α = 0.576), and the Sensory Appeal (α = 0.648), Price (α = 0.685) and Familiarity (α = 0.688) FCQ subdimensions were below the conventional 0.70 benchmark.

Bivariate associations are summarised in Table 3. The total OFCS score was not associated with the total T-FNS score (r_s_ = −0.006, *p* = 0.893), but was negatively associated with Willingness to Try New Foods (r_s_ = −0.236, *p* < 0.001) and positively associated with Trust in New Foods (r_s_ = 0.204, *p* < 0.001), both of very weak magnitude. The total FCQ score was positively associated with the total OFCS score (r_s_ = 0.395, *p* < 0.001). Among the FCQ subdimensions, Natural Content showed the strongest positive correlation with the total OFCS score (r_s_ = 0.528), followed by Health (r_s_ = 0.483) and Weight Control (r_s_ = 0.400), all *p* < 0.001, whereas Price was not significantly associated with the total OFCS score (r_s_ = 0.083, *p* = 0.062). Full correlations with the OFCS subdimensions are presented in Table 3.

Regression models for the total OFCS score are shown in Table 4. In the unadjusted models, 14.8% to 36.1% of the variance in the total OFCS score was explained, and in the age- and gender-adjusted models, 18.1% to 36.7% was explained. In Model 1, the total FCQ score was positively associated with the total OFCS score (β = 0.360, *p* < 0.001), whereas no significant association was found between the total T-FNS score and the total OFCS score (*p* > 0.05). In Model 2, Trust in New Foods (β = 0.144, *p* < 0.001), Health (β = 0.148, *p* = 0.026), Natural Content (β = 0.282, *p* < 0.001), and Weight Control (β = 0.164, *p* = 0.001) were positively associated with the total OFCS score, whereas Willingness to Try New Foods was negatively associated with the total OFCS score (β = −0.225, *p* < 0.001). No significant associations were observed between the total OFCS score and Mood, Convenience, Sensory Appeal, Price, Familiarity, or Ethical Concern (*p* > 0.05). Gender was also associated with the total OFCS score in both adjusted models, with higher scores among women, whereas age was associated with it only in Model 1, with higher scores among older participants (Table 4). Across the models, tolerance values ranged between 0.284 and 0.999 and VIF values between 1.001 and 3.523; multicollinearity was therefore not evident. Visual inspection of residual-versus-fitted plots did not indicate marked departures from linearity (Figure S1). Residual normality showed a small departure in Model 1 (Shapiro–Wilk *W* = 0.992, *p* = 0.012) but not in Model 2 (*W* = 0.996, *p* = 0.281). Heteroscedasticity was detected in both adjusted models (Koenker studentised Breusch–Pagan test, *p* = 0.008 and *p* = 0.001, respectively); however, HC3 robust standard errors did not alter the statistical significance of any model coefficient (Table S1). The maximum Cook’s distance was 0.026, and two observations had standardised residuals beyond ±3; excluding these two observations did not materially alter the estimates (largest change in β = 0.012).

Exploratory models for the six OFCS subdimension scores, adjusted for age and gender, are shown in Table 5 and Table 6; adjusted R^2^ values ranged from 0.034 (Norms, Model 1) to 0.285 (Self-Identity, Model 2). In Model 1, the total FCQ score was positively associated with all six OFCS subdimensions (β = 0.146–0.351, *p* ≤ 0.001), whereas the total T-FNS score was negatively associated only with Positive Moral Attitudes (β = −0.150, *p* < 0.001) and Information Seeking (β = −0.101, *p* = 0.015). In Model 2, Willingness to Try New Foods was negatively associated with all six OFCS subdimensions (β = −0.261 to −0.148, *p* ≤ 0.001), whereas Natural Content was positively associated with all six (β = 0.181–0.291, *p* ≤ 0.007). Trust in New Foods was positively associated with four subdimensions, Weight Control with four, and Health with three. Other associations were less consistent, while Price and Familiarity were not associated with any OFCS subdimension (Table 5 and Table 6).

## 4. Discussion

Two main patterns emerged from the analyses. First, the overall food neophobia score was not related to organic food consumption orientation, whereas its two subdimensions showed associations in opposite directions. Greater willingness to try new foods and lower trust in new foods were both associated with a stronger orientation. Second, among food choice motives, natural content showed the largest standardised association with orientation, followed by weight control and health. Together, these results suggest that dimension-specific scores may be more informative than global scores when organic food consumption orientation is examined. In relation to the research questions, organic food consumption orientation was related to the subdimensions of food neophobia rather than to the total score (RQ1), to the health, natural content and weight control motives after adjustment for age and gender (RQ2), and, for Willingness to Try New Foods and Natural Content, consistently across all six OFCS subdimensions, whereas the other associations were less consistent (RQ3). Two points extend previous work, which generally modelled food neophobia as a single construct within intention- or behaviour-oriented models [4,7,8,10,11]. The two subdimensions of the T-FNS were associated with organic food consumption orientation in opposite directions, a pattern that was not apparent in the total T-FNS score. In addition, the mutually adjusted associations of the nine FCQ motives were estimated within a single age- and gender-adjusted model using the multidimensional OFCS in a sample of adults recruited in Kırklareli, Türkiye.

The opposing directions of the two neophobia subdimension associations may partly explain why the total T-FNS score showed no relationship with organic food consumption orientation. This result does not fully align with some previous studies suggesting that food neophobia weakens the intention–behaviour relationship and reduces organic food consumption or purchase [1,4,8]. However, given that a large proportion of the participants in the present study reported consuming organic food, it is possible that organic foods were perceived by some individuals not as “new” or “unfamiliar”, but rather as “familiar” and “trustworthy”. Food neophobia has been described as a biological and behavioural protective mechanism that helps individuals avoid foods perceived as potentially harmful [35]. Therefore, the common perception of organic foods as healthier, more natural, and more reliable [36] may have directed some individuals, particularly those with lower trust in new foods, towards these products. Considering that health and natural content were among the important motivations underlying organic food consumption orientation in the present study, it is plausible that some individuals may have preferred organic foods because they perceived them as a safer option. In addition, the systematic review by Román et al. [37] suggested that individuals with higher food-related concerns may also show greater willingness to consume healthy, ecological, and organic foods.

At the same time, previous studies in the context of organic food have examined either the direct effect of food neophobia on organic food consumption or its moderating role in the attitude–intention or intention–behaviour relationship, and the findings have been inconsistent. Some studies have reported that food neophobia did not predict willingness to try organic foods [5], was not associated with preference for organic foods [6], or did not play a moderating role in the attitude–intention relationship [7]. In contrast, other studies have shown that food neophobia negatively affected intention to purchase organic food [8] and acted as a negative moderator of the intention–behaviour relationship [4]. Rehman et al. [11] reported that food neophobia reduced satisfaction, which in turn weakened organic food purchase intention, whereas Kashif et al. [10] found that food neophobia positively moderated the intention–behaviour relationship. These inconsistent findings may be explained by differences in sociocultural context, economic conditions, market size, familiarity with products, lifestyle characteristics, and individual perceptions of organic food. In this respect, the present findings suggest that food neophobia may relate to organic food consumption orientation differently across its subdimensions. This pattern should nevertheless be interpreted cautiously given the modest internal consistency of the Trust in New Foods subscale.

The present study also found that health, natural content, and weight control motives were positively associated with organic food consumption orientation. This finding suggests that personal (egoistic) motives may play a more central role in organic food consumption orientation among the participants. Similarly, a growing body of literature has shown that consumers’ perceptions, attitudes, intentions, and behaviours related to organic foods are more strongly influenced by personal or egoistic motives, such as health concerns and safety, than by altruistic motives, such as environmental, social, and animal welfare concerns [3,12,38,39,40,41,42]. Moreover, health and safety concerns are thought to be more dominant in developing countries, whereas environmental, social, and ethical concerns may be more salient in developed countries [13,43]. In the Turkish context, the findings of the present study are consistent with this pattern.

A positive association was also observed for the health motive, although its standardised coefficient was smaller than that for natural content. This pattern is broadly consistent with previous evidence linking health-related beliefs, perceived health benefits and health consciousness to attitudes, intentions and behaviours towards organic foods [12,15,36,39,40,41]. Health concerns have also been described as a universal driver of organic food consumption. Organic foods are widely perceived as free from preservatives, pesticide residues, artificial additives, synthetic fertilisers and genetically modified organisms, and this perception may lead individuals to choose them to protect their health [13]. Consumers thus appear to equate the healthiness of organic food largely with its naturalness, that is, with the belief that it is unpolluted by additives, preservatives or excessive human intervention [44].

Natural content emerged as the food choice motive most strongly related to organic food consumption orientation, in line with this literature. Perceived naturalness has previously been linked to organic food preference [45], attitudes towards organic food [46] and purchase intention [47]. Although its relative weight varies between studies, the absence of artificial additives, chemicals, pesticides and genetically modified ingredients has also been reported as a factor shaping consumer preferences for organic food [2,8,12,36,41,48,49].

The present study further showed that weight control motivation was an important factor associated with organic food consumption orientation. This finding may be related to the cognitive bias referred to in the literature as the “organic halo effect”, whereby organic foods are perceived as healthier, lower in calories, lower in fat, and higher in fibre than their non-organic counterparts [50,51]. However, this perception may also carry potential health risks, as it may lead individuals to underestimate calorie content and to believe that even unhealthy organic products can be consumed more frequently [50]. On the other hand, recent systematic reviews and meta-analyses have suggested that organic food consumption may be associated not only with a range of health benefits but also with a lower risk of overweight and obesity [52,53,54]. The role of weight-related motives in organic food consumption has been investigated less extensively. For example, some studies have reported that weight control motivation is not associated with increased organic food consumption [55] and does not predict attitudes towards organic food [7]. Łopacińska [40] noted that only a small proportion of participants preferred organic foods for aesthetic reasons and that concerns related to appearance were viewed not as a primary motivation but rather as a secondary outcome of healthy living. In contrast, Oroian et al. [56] identified weight concern as one of the main reasons for consuming organic food. Consumers with health-related concerns may be particularly interested in the effects of organic foods on weight control and health. Similarly, in the qualitative study by Rodman et al. [57], some participants attributed weight gain to chemicals, hormones, and steroids found in non-organic foods. The authors suggested that this perception may strengthen the belief that an organic diet can alleviate weight-related concerns. The present study measured food choice motives rather than dietary intake or body weight. The association observed here may therefore partly reflect this halo effect rather than an established nutritional advantage. Testing it would require studies that measure organic food intake together with dietary and anthropometric outcomes.

Overall, the inconsistencies across studies may reflect not only methodological differences but also the complex and multidimensional nature of individual food choice. Consumers’ habits, perceptions, attitudes, and behaviours may vary according to culture, geographic region, socioeconomic conditions, and the level of market development. In addition, the attitude–intention–behaviour gap, which has frequently been emphasised in the context of organic food [3], may also contribute to the diversity of findings in this field. These findings may help identify motivational correlates of organic food consumption orientation, although prospective and experimental studies are required before implications for dietary interventions can be established.

### Limitations and Future Directions

Several features of the study constrain interpretation. Because predictors and outcomes were measured at the same time, their temporal ordering cannot be established. The convenience sample was recruited in the city centre of Kırklareli and differed from the 2023 provincial population aged 18–64 years in several respects. The sample contained a higher proportion of women (61.3% versus 48.4%) and of adults under 30 years of age (34.2% versus 25.0%), a lower proportion of adults aged 40–64 years (36.8% versus 53.8%), and a higher proportion of participants who had completed higher education (54.3% versus 24.5%) [17,58]. This comparison is descriptive; because recruitment was confined to the city centre and used convenience sampling, it does not establish representativeness. The findings therefore describe a predominantly urban sample consisting mainly of women and younger adults and should not be regarded as representative of adults in Kırklareli, in Türkiye or in other sociocultural settings. All key variables, including body weight and height, were self-reported and collected in a single session, which creates the possibility of recall, social desirability and common method biases. The OFCS reflects orientation towards organic food rather than verified purchasing or intake. Although 73.5% of participants reported consuming organic food, reported consumption was not modelled as an outcome, and participants’ own definitions of organic food may have introduced misclassification. Residual confounding remains possible because the adjusted models included only age and gender, which were selected a priori, and not education, income or employment status. Finally, the exploratory analyses of the OFCS subdimensions involved a large number of comparisons, so associations with *p* values close to 0.05 warrant cautious interpretation.

Measurement precision also deserves comment. In the present sample, the internal consistency of the T-FNS (Cronbach’s alpha 0.643) was lower than in the Turkish adaptation study (0.805), and its two subdimensions were only weakly intercorrelated (r_s_ = 0.150, *p* = 0.001). Some FCQ subdimensions also fell below 0.70, as reported for two subdimensions in the adaptation study [26]. Findings involving these scales should therefore be read with caution. Future studies should use longitudinal designs to clarify temporal relationships and incorporate more objective measures such as purchase records, consumer panels, observational data or measured anthropometry. Additional determinants, including knowledge and awareness, environmental values, trust in certification and labelling, socioeconomic factors, social media influence and food neophilia, also merit examination.

## 5. Conclusions

Overall, organic food consumption orientation was unrelated to the global food neophobia score but varied with specific neophobia dimensions and selected food choice motives. Greater willingness to try unfamiliar foods, lower trust in new foods, and stronger natural content, weight control and health motives were each associated with higher OFCS scores. These findings suggest that specific psychosocial dimensions may be more informative than global food neophobia or food choice scores for understanding consumer orientations towards organic food.

## Figures and Tables

**Table 1:** Distribution of the participants’ descriptive characteristics (*n* = 506).

Variables	*n*	%
**Gender**		
Men	196	38.7
Women	310	61.3
**Age (years), mean ± SD: 36.47 ± 12.05; median (Q1–Q3): 35 (26–45); min: 18, max: 64**		
18–29	173	34.2
30–39	147	29.1
40–64	186	36.8
**Place of residence**		
Urban	488	96.4
Rural	18	3.6
**Marital status**		
Single	217	42.9
Married	289	57.1
**Educational level**		
Primary school	29	5.7
Middle school	134	26.5
High school	68	13.4
Undergraduate	249	49.2
Postgraduate	26	5.1
**Employment status**		
Unemployed/Not working	169	33.4
Employed	337	66.6
**Occupation**		
Civil servant	168	33.2
Self-employed	43	8.5
Worker	126	24.9
Unemployed	136	26.9
Retired	33	6.5
**Income level**		
Insufficient	131	25.9
Sufficient	375	74.1
**Chronic disease**		
No	306	60.5
Yes	200	39.5
**Smoking status**		
Yes	169	33.4
No	278	54.9
Former smoker	59	11.7
**Alcohol use**		
Yes	148	29.2
No	358	70.8
**Regular exercise**		
Yes	103	20.4
No	403	79.6
**Previous dieting**		
Yes	166	32.8
No	340	67.2
**BMI**		
Underweight	21	4.2
Normal	258	51.0
Overweight	164	32.4
Obese	63	12.5
**Received nutrition education**		
Yes	87	17.2
No	419	82.8
**Perceived sufficient knowledge about organic food**		
Yes	221	43.7
No	285	56.3
**Do you consume organic food?**		
Yes	372	73.5
No	134	26.5
**Frequency of organic food consumption**		
More than once a week	188	37.2
Once a week	100	19.8
2–3 times a month	41	8.1
Once a month	43	8.5
I do not consume	134	26.5
How do you distinguish organic foods from other products when purchasing? *		
Presence of an organic food label/logo	274	54.2
Packaged product	55	10.9
Displayed separately from other products	128	25.3
No specific criterion	136	26.9
**Self-perceived healthy eating**		
Yes	158	31.2
No	146	28.9
Undecided/I do not know	202	39.9

* Multiple responses were allowed; percentages therefore exceed 100. SD, standard deviation; Q1, first quartile; Q3, third quartile. BMI, body mass index; BMI categories follow the World Health Organization classification. Employment status distinguishes participants in paid work from those not in paid work, including retired participants; occupation gives the type of work. Variable names are shown in bold.

**Table 2:** Total and subdimension scores of the T-FNS, FCQ and OFCS (*n* = 506).

Scale and Subdimensions	*n*	Number of Items	Mean ± SD	Median (Q1–Q3)	Min–Max	Cronbach’s Alpha
**Food Neophobia Scale (T-FNS)**						
Total	506	10	40.78 ± 8.81	40 (35–47)	17–67	0.643
Willingness to Try New Foods	506	4	16.25 ± 5.50	16 (12–20)	4–28	0.742
Trust in New Foods	506	6	24.53 ± 6.09	24 (20–29)	9–39	0.576
**Food Choice Questionnaire (FCQ)**						
Total	506	36	97.07 ± 17.33	93.5 (86–110)	46–132	0.939
Health	506	6	17.67 ± 4.19	18 (15–21)	6–24	0.855
Mood	506	6	17.48 ± 4.07	18 (15–21)	6–24	0.828
Convenience	506	5	14.79 ± 3.49	15 (12–18)	5–20	0.799
Sensory Appeal	506	4	12.93 ± 2.38	13 (11–15)	5–16	0.648
Natural Content	506	3	8.97 ± 2.56	9 (7–11)	3–12	0.853
Price	506	3	9.09 ± 2.12	9 (7–11)	3–12	0.685
Weight Control	506	3	8.08 ± 2.47	8 (6–10)	3–12	0.785
Familiarity	506	3	8.42 ± 2.26	8 (7–10)	3–12	0.688
Ethical Concern	506	3	7.72 ± 2.63	7 (6–10)	3–12	0.780
**Organic Food Consumption Scale (OFCS)**						
Total	506	18	61.62 ± 12.20	63 (55–70)	27–90	0.925
Norms	506	4	11.23 ± 3.74	12 (8–13)	4–20	0.890
Health Consciousness	506	3	11.12 ± 2.35	11 (9–12)	3–15	0.828
Self-Identity	506	3	9.99 ± 2.72	10 (8–12)	3–15	0.844
Benefits of Organic Food Consumption	506	4	15.35 ± 3.25	16 (14–17)	5–20	0.901
Positive Moral Attitudes	506	2	7.29 ± 1.96	8 (6–8)	2–10	0.890
Information Seeking	506	2	6.65 ± 2.32	7 (5–8)	2–10	0.924

SD, standard deviation; Q1, first quartile; Q3, third quartile; T-FNS, Food Neophobia Scale (Turkish version); FCQ, Food Choice Questionnaire; OFCS, Organic Food Consumption Scale. Higher scores on both T-FNS subdimensions indicate a stronger tendency to avoid new foods; that is, a higher Willingness to Try New Foods score indicates lower willingness and a higher Trust in New Foods score indicates lower trust. Scale names are shown in bold.

**Table 3:** Correlations between the total and subdimension scores of the OFCS and those of the T-FNS and FCQ (*n* = 506).

	Organic Food Consumption Scale (OFCS)													
	Total		Norms		Health Consciousness		Self-Identity		Benefits of Organic Food Consumption		Positive Moral Attitudes		Information Seeking	
	*r_s_*	*p*	*r_s_*	*p*	*r_s_*	*p*	*r_s_*	*p*	*r_s_*	*p*	*r_s_*	*p*	*r_s_*	*p*
**Food Neophobia Scale (T-FNS)**														
Total	−0.006	0.893	−0.004	0.934	0.085	0.055	0.024	0.588	0.007	0.880	−0.092	0.038 *	−0.047	0.292
Willingness to Try New Foods	−0.236	<0.001 ***	−0.154	0.001 **	−0.105	0.018 *	−0.185	<0.001 ***	−0.175	<0.001 ***	−0.287	<0.001 ***	−0.173	<0.001 ***
Trust in New Foods	0.204	<0.001 ***	0.121	0.006 **	0.212	<0.001 ***	0.210	<0.001 ***	0.170	<0.001 ***	0.118	0.008 **	0.094	0.035 *
**Food Choice Questionnaire (FCQ)**														
Total	0.395	<0.001 ***	0.165	<0.001 ***	0.281	<0.001 ***	0.351	<0.001 ***	0.313	<0.001 ***	0.376	<0.001 ***	0.379	<0.001 ***
Health	0.483	<0.001 ***	0.204	<0.001 ***	0.350	<0.001 ***	0.420	<0.001 ***	0.410	<0.001 ***	0.439	<0.001 ***	0.425	<0.001 ***
Mood	0.222	<0.001 ***	0.038	0.390	0.191	<0.001 ***	0.223	<0.001 ***	0.132	0.003 **	0.223	<0.001 ***	0.273	<0.001 ***
Convenience	0.093	0.037 *	0.032	0.475	0.022	0.625	0.055	0.219	0.071	0.113	0.171	<0.001 ***	0.113	0.011 *
Sensory Appeal	0.182	<0.001 ***	0.019	0.677	0.180	<0.001 ***	0.101	0.023 *	0.156	<0.001 ***	0.186	<0.001 ***	0.271	<0.001 ***
Natural Content	0.528	<0.001 ***	0.298	<0.001 ***	0.361	<0.001 ***	0.426	<0.001 ***	0.464	<0.001 ***	0.421	<0.001 ***	0.445	<0.001 ***
Price	0.083	0.062	0.011	0.810	0.071	0.109	0.075	0.090	0.093	0.037 *	0.125	0.005 **	0.084	0.060
Weight Control	0.400	<0.001 ***	0.234	<0.001 ***	0.298	<0.001 ***	0.357	<0.001 ***	0.309	<0.001 ***	0.354	<0.001 ***	0.312	<0.001 ***
Familiarity	0.325	<0.001 ***	0.153	0.001 **	0.255	<0.001 ***	0.299	<0.001 ***	0.223	<0.001 ***	0.300	<0.001 ***	0.282	<0.001 ***
Ethical Concern	0.363	<0.001 ***	0.251	<0.001 ***	0.169	<0.001 ***	0.351	<0.001 ***	0.241	<0.001 ***	0.349	<0.001 ***	0.328	<0.001 ***

Spearman correlation analysis. r_s_, Spearman rank correlation coefficient. * *p* < 0.05, ** *p* < 0.01, *** *p* < 0.001. Higher scores on both T-FNS subdimensions indicate a stronger tendency to avoid new foods. T-FNS, Food Neophobia Scale (Turkish version); FCQ, Food Choice Questionnaire; OFCS, Organic Food Consumption Scale. Scale names are shown in bold.

**Table 4:** Multivariable linear regression analysis of the associations between the total OFCS score and the total and subdimension scores of the T-FNS and FCQ (*n* = 506).

Predictors	Unadjusted					Adjusted ^1^				
	B	SE	95% CI	β	*p*	B	SE	95% CI	β	*p*
**Model 1**										
T-FNS Total	−0.036	0.057	−0.147, 0.076	−0.026	0.532	−0.082	0.057	−0.194, 0.030	−0.060	0.149
FCQ Total	0.274	0.029	0.217, 0.331	0.389	<0.001 ***	0.253	0.029	0.197, 0.310	0.360	<0.001 ***
	Adj. R^2^ = 0.148; *F* = 44.930 ***					Adj. R^2^ = 0.181; *F* = 28.878 ***				
**Model 2**										
Willingness to Try New Foods	−0.471	0.082	−0.633, −0.310	−0.212	<0.001 ***	−0.500	0.083	−0.664, −0.337	−0.225	<0.001 ***
Trust in New Foods	0.303	0.078	0.150, 0.455	0.151	<0.001 ***	0.288	0.078	0.136, 0.441	0.144	<0.001 ***
Health	0.434	0.194	0.054, 0.814	0.149	0.025 *	0.430	0.193	0.051, 0.810	0.148	0.026 *
Mood	−0.275	0.174	−0.617, 0.066	−0.092	0.114	−0.267	0.174	−0.608, 0.074	−0.089	0.125
Convenience	−0.166	0.181	−0.522, 0.190	−0.048	0.359	−0.200	0.183	−0.560, 0.159	−0.057	0.274
Sensory Appeal	−0.092	0.257	−0.596, 0.413	−0.018	0.722	−0.101	0.256	−0.604, 0.402	−0.020	0.693
Natural Content	1.445	0.268	0.919, 1.971	0.303	<0.001 ***	1.343	0.269	0.814, 1.873	0.282	<0.001 ***
Price	−0.295	0.281	−0.847, 0.257	−0.051	0.294	−0.109	0.290	−0.679, 0.462	−0.019	0.708
Weight Control	0.880	0.249	0.392, 1.369	0.178	<0.001 ***	0.807	0.249	0.317, 1.297	0.164	0.001 **
Familiarity	0.042	0.274	−0.495, 0.580	0.008	0.877	0.028	0.275	−0.512, 0.568	0.005	0.919
Ethical Concern	0.215	0.231	−0.238, 0.668	0.046	0.352	0.203	0.230	−0.249, 0.656	0.044	0.378
	Adj. R^2^ = 0.361; *F* = 26.927 ***					Adj. R^2^ = 0.367; *F* = 23.539 ***				

^1^ Adjusted for age and gender; gender was coded as 1 = man and 2 = woman. In the adjusted models, the covariate coefficients were: Model 1, age B = 0.104, SE = 0.042, 95% CI 0.022, 0.186, β = 0.103, *p* = 0.013, and gender B = 4.318, SE = 1.026, 95% CI 2.303, 6.333, β = 0.173, *p* < 0.001; Model 2, age B = 0.048, SE = 0.038, 95% CI −0.028, 0.123, β = 0.047, *p* = 0.216, and gender B = 2.345, SE = 0.957, 95% CI 0.465, 4.226, β = 0.094, *p* = 0.015. B, unstandardised regression coefficient; SE, conventional ordinary least-squares standard error of B; the 95% CIs and *p* values reported in the main table are also based on conventional OLS standard errors. Because heteroscedasticity was detected in both adjusted models, HC3 heteroscedasticity-consistent standard errors, 95% CIs and *p* values were calculated as a sensitivity analysis; these did not alter the statistical significance of any model coefficient (Table S1). CI, confidence interval; β, standardised regression coefficient; Adj. R^2^, adjusted R-square. * *p* < 0.05, ** *p* < 0.01, *** *p* < 0.001. Higher scores on both T-FNS subdimensions indicate a stronger tendency to avoid new foods. T-FNS, Food Neophobia Scale (Turkish version); FCQ, Food Choice Questionnaire; OFCS, Organic Food Consumption Scale. Model headings are shown in bold.

**Table 5:** Multivariable linear regression analysis of the associations between the Norms, Health Consciousness and Self-Identity subdimensions of the OFCS and the total and subdimension scores of the T-FNS and FCQ (*n* = 506).

Predictors	Norms			Health Consciousness			Self-Identity		
	β	SE	*p*	β	SE	*p*	β	SE	*p*
**Model 1**									
T-FNS Total	−0.045	0.045	0.315	0.020	0.043	0.640	0.013	0.043	0.764
FCQ Total	0.146	0.044	0.001 **	0.254	0.043	<0.001 ***	0.326	0.042	<0.001 ***
Age	0.083	0.045	0.065	0.121	0.043	0.005 **	−0.016	0.043	0.711
Gender	0.097	0.045	0.029 *	0.122	0.043	0.005 **	0.077	0.043	0.073
Adj. R^2^	0.034			0.100			0.112		
*F*	5.417 ***			15.006 ***			16.893 ***		
**Model 2**									
Willingness to Try New Foods	−0.148	0.044	0.001 **	−0.149	0.042	<0.001 ***	−0.164	0.040	<0.001 ***
Trust in New Foods	0.089	0.045	0.0498 *	0.150	0.044	0.001 **	0.175	0.041	<0.001 ***
Health	−0.067	0.078	0.391	0.145	0.075	0.053	0.145	0.071	0.041 *
Mood	−0.159	0.068	0.020 *	0.048	0.065	0.465	0.050	0.062	0.416
Convenience	0.007	0.061	0.915	−0.123	0.059	0.037 *	−0.070	0.056	0.206
Sensory Appeal	−0.040	0.058	0.494	0.036	0.056	0.518	−0.182	0.053	0.001 **
Natural Content	0.181	0.066	0.007 **	0.191	0.064	0.003 **	0.195	0.060	0.001 **
Price	−0.049	0.059	0.410	0.054	0.057	0.345	−0.027	0.054	0.613
Weight Control	0.175	0.059	0.003 **	0.114	0.057	0.046 *	0.161	0.054	0.003 **
Familiarity	0.022	0.060	0.718	−0.014	0.057	0.803	0.044	0.054	0.413
Ethical Concern	0.150	0.058	0.011 *	−0.152	0.056	0.007 **	0.069	0.053	0.190
Age	0.029	0.045	0.511	0.103	0.043	0.016 *	−0.066	0.040	0.101
Gender	0.043	0.045	0.336	0.080	0.043	0.063	0.004	0.041	0.926
Adj. R^2^	0.129			0.196			0.285		
*F*	6.746 ***			10.445 ***			16.514 ***		

Model 1 includes the total T-FNS and FCQ scores and Model 2 the T-FNS and FCQ subdimension scores; all models were adjusted for age and gender; gender was coded as 1 = man and 2 = woman. β, standardised regression coefficient; SE, standard error of the standardised coefficient; Adj. R^2^, adjusted R-square. * *p* < 0.05, ** *p* < 0.01, *** *p* < 0.001. Higher scores on both T-FNS subdimensions indicate a stronger tendency to avoid new foods. T-FNS, Food Neophobia Scale (Turkish version); FCQ, Food Choice Questionnaire; OFCS, Organic Food Consumption Scale. Model headings are shown in bold.

**Table 6:** Multivariable linear regression analysis of the associations between the Benefits of Organic Food Consumption, Positive Moral Attitudes and Information Seeking subdimensions of the OFCS and the total and subdimension scores of the T-FNS and FCQ (*n* = 506).

Predictors	Benefits of Organic Food Consumption			Positive Moral Attitudes			Information Seeking		
	β	SE	*p*	β	SE	*p*	β	SE	*p*
**Model 1**									
T-FNS Total	−0.035	0.043	0.411	−0.150	0.042	<0.001 ***	−0.101	0.041	0.015 *
FCQ Total	0.269	0.042	<0.001 ***	0.345	0.041	<0.001 ***	0.351	0.041	<0.001 ***
Age	0.084	0.043	0.051	0.083	0.042	0.048 *	0.118	0.041	0.004 **
Gender	0.180	0.043	<0.001 ***	0.122	0.042	0.003 **	0.182	0.041	<0.001 ***
Adj. R^2^	0.117			0.159			0.184		
*F*	17.672 ***			24.926 ***			29.434 ***		
**Model 2**									
Willingness to Try New Foods	−0.159	0.040	<0.001 ***	−0.261	0.040	<0.001 ***	−0.161	0.041	<0.001 ***
Trust in New Foods	0.126	0.041	0.003 **	0.059	0.041	0.153	0.029	0.042	0.494
Health	0.226	0.071	0.002 **	0.121	0.071	0.089	0.151	0.073	0.038 *
Mood	−0.175	0.062	0.005 **	−0.113	0.062	0.069	0.022	0.063	0.734
Convenience	−0.074	0.056	0.184	0.044	0.056	0.436	−0.037	0.057	0.523
Sensory Appeal	0.047	0.053	0.383	−0.020	0.053	0.701	0.090	0.055	0.099
Natural Content	0.291	0.061	<0.001 ***	0.183	0.060	0.003 **	0.205	0.062	0.001 **
Price	0.034	0.054	0.532	−0.058	0.054	0.286	−0.042	0.055	0.446
Weight Control	0.097	0.054	0.074	0.151	0.054	0.005 **	0.010	0.055	0.854
Familiarity	−0.061	0.055	0.262	0.050	0.054	0.359	−0.001	0.056	0.982
Ethical Concern	−0.037	0.053	0.485	0.086	0.053	0.105	0.041	0.054	0.447
Age	0.038	0.041	0.348	0.040	0.041	0.321	0.086	0.042	0.040 *
Gender	0.116	0.041	0.005 **	0.058	0.041	0.159	0.126	0.042	0.003 **
Adj. R^2^	0.273			0.278			0.240		
*F*	15.580 ***			15.926 ***			13.255 ***		

Model 1 includes the total T-FNS and FCQ scores and Model 2 the T-FNS and FCQ subdimension scores; all models were adjusted for age and gender; gender was coded as 1 = man and 2 = woman. β, standardised regression coefficient; SE, standard error of the standardised coefficient; Adj. R^2^, adjusted R-square. * *p* < 0.05, ** *p* < 0.01, *** *p* < 0.001. Higher scores on both T-FNS subdimensions indicate a stronger tendency to avoid new foods. T-FNS, Food Neophobia Scale (Turkish version); FCQ, Food Choice Questionnaire; OFCS, Organic Food Consumption Scale. Model headings are shown in bold.

## Data Availability

The datasets generated and analysed during the current study are not publicly available due to confidentiality and ethical restrictions, but are available from the corresponding author on reasonable request.

## Supplementary Materials

The following supporting information can be downloaded at: https://www.mdpi.com/article/10.3390/foods15203637/s1, Figure S1. Regression diagnostic plots for the age- and gender-adjusted multivariable linear regression models of the total Organic Food Consumption Scale (OFCS) score (*n* = 506): (a) residual-versus-fitted plot for Model 1; (b) normal Q–Q plot of the standardised residuals for Model 1; (c) residual-versus-fitted plot for Model 2; (d) normal Q–Q plot of the standardised residuals for Model 2. Model 1 includes the total Food Neophobia Scale (T-FNS) and Food Choice Questionnaire (FCQ) scores as predictors; Model 2 includes the T-FNS and FCQ subdimension scores. Shapiro–Wilk test for residual normality: Model 1, *W* = 0.992, *p* = 0.012; Model 2, *W* = 0.996, *p* = 0.281. Table S1. Sensitivity analysis with HC3 heteroscedasticity-consistent standard errors for the age- and gender-adjusted multivariable linear regression models of the total OFCS score (*n* = 506).
