# Supplementary material for: Associations of Food Neophobia and Food Choice Motives with Organic Food Consumption Orientation Among Adults in Türkiye: A Cross-Sectional Study
Source: Foods. 2026 Oct 13;15(20):3637. doi: 10.3390/foods15203637 (PMC13703729; doi:10.3390/foods15203637)
Supplement: Supplementary file 1 [file foods-15-03637-s001.zip › foods-4592542-supplementary.pdf]

Supplementary Materials

Associations of Food Neophobia and Food Choice Motives with Organic Food Consumption Orientation Among Adults in Türkiye: A Cross-Sectional Study

Muhammet Ali Çakır, Merve Pehlivan, Neslişah Denkçi and Perim Fatma Türker

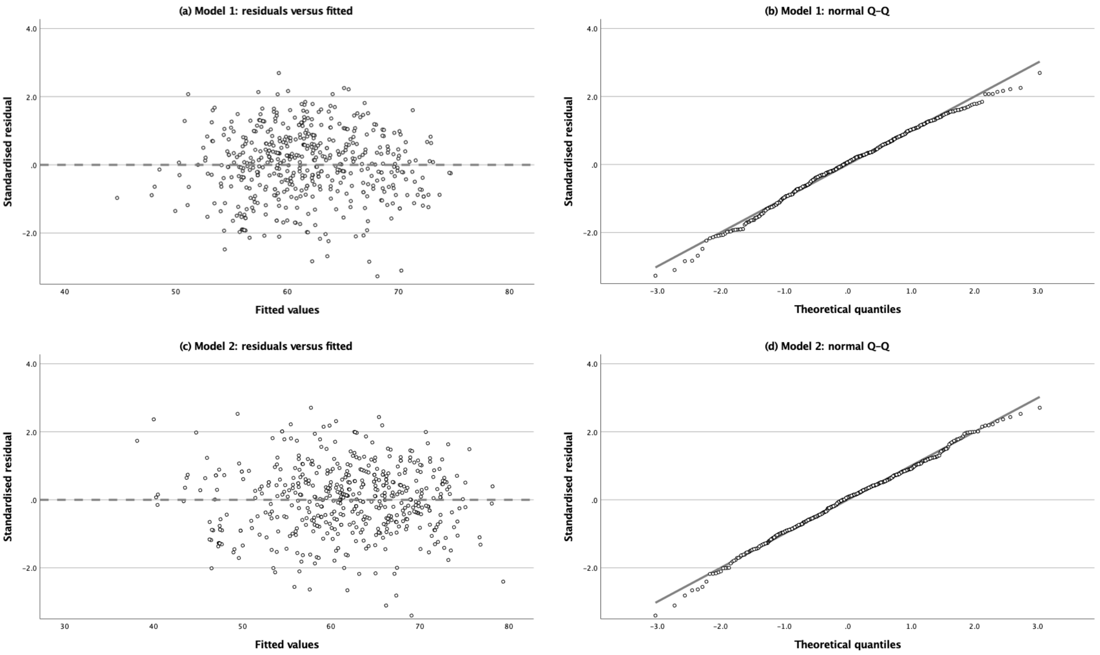

Figure S1. Regression diagnostic plots for the age- and gender-adjusted multivariable linear regression models of the total Organic Food Consumption Scale (OFCS) score (n = 506): (a) residual-versus-fitted plot for Model 1; (b) normal Q–Q plot of the standardised residuals for Model 1; (c) residual-versus-fitted plot for Model 2; (d) normal Q–Q plot of the standardised residuals for Model 2. Model 1 includes the total Food Neophobia Scale (T-FNS) and Food Choice Questionnaire (FCQ) scores as predictors; Model 2 includes the T-FNS and FCQ subdimension scores. Shapiro–Wilk test for residual normality: Model 1, W = 0.992, p = 0.012; Model 2, W = 0.996, p = 0.281.

Table S1. Sensitivity analysis with HC3 heteroscedasticity-consistent standard errors for the age- and gender-adjusted multivariable linear regression models of the total OFCS score (n = 506).

Predictors	B	HC3 SE	95% CI	β	p
**Model 1**					
T-FNS Total	−0.082	0.053	−0.187, 0.022	−0.060	0.123
FCQ Total	0.253	0.029	0.196, 0.311	0.360	<0.001***
Age	0.104	0.043	0.019, 0.189	0.103	0.016*
Gender	4.318	1.096	2.164, 6.472	0.173	<0.001***
	Adj. R^2 = 0.181				
**Model 2**					
Willingness to Try New Foods	−0.500	0.096	−0.689, −0.311	−0.225	<0.001***
Trust in New Foods	0.288	0.084	0.123, 0.454	0.144	<0.001***
Health	0.430	0.211	0.015, 0.845	0.148	0.042*
Mood	−0.267	0.176	−0.612, 0.079	−0.089	0.130
Convenience	−0.200	0.177	−0.549, 0.148	−0.057	0.260
Sensory Appeal	−0.101	0.246	−0.585, 0.383	−0.020	0.682
Natural Content	1.343	0.276	0.802, 1.885	0.282	<0.001***
Price	−0.109	0.299	−0.696, 0.479	−0.019	0.716
Weight Control	0.807	0.249	0.318, 1.296	0.164	0.001**
Familiarity	0.028	0.268	−0.498, 0.554	0.005	0.916
Ethical Concern	0.203	0.234	−0.257, 0.663	0.044	0.386
Age	0.048	0.043	−0.037, 0.132	0.047	0.269
Gender	2.345	0.981	0.417, 4.274	0.094	0.017*
	Adj. R^2 = 0.367				

B, unstandardised regression coefficient; HC3 SE, heteroscedasticity-consistent (HC3) standard error of B; CI, 95% confidence interval based on the HC3 standard error and the t distribution with the residual degrees of freedom (501 for Model 1 and 492 for Model 2); β, standardised regression coefficient; p, two-sided p value based on the HC3 standard error. * $p < 0.05$, ** $p < 0.01$, *** $p < 0.001$. B and β are identical to those reported in Table 4 of the main text; only the standard errors, confidence intervals and p values differ. Gender was coded as 1 = man and 2 = woman. Higher scores on both T-FNS subdimensions indicate a stronger tendency to avoid new foods. Adj. R^2, adjusted R-square. The statistical significance of every model coefficient was the same as with conventional standard errors (Table 4).
